# Impact of central obesity on esophageal motility and mucosal barrier function based on conventional CT evaluation

**DOI:** 10.3389/fmed.2026.1768926

**Published:** 2026-03-24

**Authors:** Jiang Guidong, Zhang Qinhe, Sun Yinan, Wang Hongkai, Liu Ailian, Duan Zhijun

**Affiliations:** 1 The First Affiliated Hospital of DaLian Medical University, Dalian, China; 2 Department of Radiology, The First Affiliated Hospital of Dalian Medical University, Dalian, China; 3 Technology Innovation Center of Hyperpolarized MRI, Dalian, China; 4 Technology Innovation Center of Artificial Intelligence in Medical Imaging, Dalian, China; 5 Faculty of Medicine, School of Biomedical Engineering, Dalian University of Technology, Dalian, China; 6 Department of Gastroenterology, The First Affiliated Hospital of DaLian Medical University, Dalian, China

**Keywords:** 24h pH-impedance, central obesity, computed tomography, esophageal funciton, high-resolution manometry

## Abstract

**Objective:**

The study aims to automatically quantify visceral and subcutaneous adipose tissue content using computed tomography (CT) for assessing central obesity severity, and to investigate the impact of central obesity on esophageal motility and mucosal barrier function.

**Methods:**

This retrospective study included patients who underwent fiberoptic gastroduodenoscopy, high-resolution manometry (HRM), 24-h pH-impedance monitoring, and non-contrast abdominal CT. Participants were stratified into quartiles (Q1–Q4) based on their visceral-to-subcutaneous fat area ratio (V/S), calculated using the TotalSegmentator tool. We compared baseline characteristics, HRM parameters and 24-h pH-impedance parameters across groups. Regression and correlation analyses were performed to evaluate associations between V/S and esophageal motility/mucosal barrier function.

**Results:**

Of 185 patients stratified by V/S, Group Q1 (V/S ≤ 1.01, *n* = 48) had mean age 52.75 years and median BMI 24.30 kg/m^2^; Q2 (1.02 < V/S < 1.29, *n* = 45): age 56.62 years, BMI 25.95 kg/m^2^; Q3 (1.30 < V/S < 1.75, *n* = 46): age 58.43 years, BMI 24.11 kg/m^2^; Q4 (V/S ≥ 1.76, *n* = 46): age 60.65 years, BMI 25.67 kg/m^2^. Gastroesophageal reflux disease (GERD) prevalence increased across groups (22.92, 31.11, 34.78, 54.35%). Significant intergroup differences (all *P* < 0.05) were found in gender, GERD prevalence, lower esophageal sphincter (LES) resting pressure, esophagogastric junction contractile integral (EGJ-CI), acid exposure time (AET), DeMeester score (DMS), and mean nocturnal baseline impedance (MNBI) Z3-Z6. After adjusting for Hiatal Hernia, EGJ type, Mean Acid Clearance Time, and Bolus Clearance Time (BCT) in the linear regression model, V/S was positively correlated with LES resting pressure, EGJ-CI, AET, and DMS, but negatively with MNBI Z3-Z6. Decreased MNBI was primarily associated with acid reflux across all groups, while additional correlations with LES resting pressure and EGJ-CI were observed specifically in groups Q1, Q3, and Q4. The strongest associations were seen in group Q4.

**Conclusion:**

Central obesity significantly disrupts esophageal motility and weakens the esophageal mucosal barrier, thereby precipitating mucosal injury. Varying degrees of central obesity can further impair the integrity of esophageal mucosa by inducing esophageal motility disorders or acid reflux.

## 1 Introduction

With the improvement of living standards, more than one billion people worldwide are currently plagued by obesity. Between 1990 and 2022, the obesity rate among adults has more than doubled, while the obesity rate among children and adolescents has even more than tripled (1). Obesity is associated with digestive system diseases, metabolic diseases, cardiovascular diseases, obstructive sleep apnea, and various cancers (breast, colorectal, liver, gallbladder, gastric cardia, esophageal, and pancreatic), and has emerged as a major global health concern (2). Obesity, particularly central obesity, is a primary risk factor for reflux esophagitis (RE) (3, 4). Obesity can impair the structural and functional integrity of the esophageal mucosa, and this damage is independent of gastroesophageal reflux (GER) (5, 6). Additionally, obesity may induce esophageal motor disorders (7, 8).

Body mass index (BMI) is commonly used as an indicator for assessing obesity; yet, it only reflects general obesity. With advancing age, as fat mass increases while muscle mass decreases, BMI fails to intuitively reflect changes in abdominal fat distribution and muscle mass (9). Currently, the gold standard for assessing abdominal fat distribution is medical imaging technology, particularly computed tomography (CT). It offers short detection time, broader applicability to populations, and relatively high clinical popularity, enabling quantitative evaluation of visceral adipose tissue (VAT) and subcutaneous adipose tissue (SAT) (10). Studies have confirmed that central obesity induced by increased VAT is strongly associated with RE (11, 12), and central obesity is also closely linked to Barrett’s esophagus (BE) and esophageal adenocarcinoma (13). VAT exhibits high metabolic activity and secretes a variety of substances. These substances participate in signal transduction through hormonal and inflammatory pathways, leading to systemic metabolic dysfunction. In contrast, SAT has low metabolic activity, serving as a fat storage depot, and exerts a protective effect against cardiometabolic diseases and other conditions (14). Additional studies have demonstrated that a high visceral-to-subcutaneous adipose tissue ratio (V/S) is an independent risk factor for erosive esophagitis and reflux symptoms (15, 16).

Studies have demonstrated that obesity can induce alterations in esophageal pressure, including lower esophageal sphincter (LES) dysfunction, elevated LES pressure, diffuse esophageal spasm, nutcracker esophagus and ineffective esophageal motility (17–19). Yet, the correlation between BMI and the development of GERD as well as esophageal dysfunction remains controversial. Jaffin et al. (18) conducted a study on 111 patients with a BMI ≥ 40 kg/m^2^, and the results showed that 61% of obese individuals had abnormal esophageal manometry findings. Yet, this study did not identify a correlation between BMI and reduced LES pressure, and morbidly obese patients with low LES pressure did not present with symptoms related to GER. This is because an elevated BMI only indicates an increase in general obesity, including increased VAT (which can induce metabolic abnormalities) and SAT (a neutral fat depot). Thus, the occurrence of esophageal dysfunction in patients with general obesity may be caused by VAT, a factor not fully captured by BMI measurements. Currently, there are relatively few studies investigating the impact of VAT on esophageal function.

Therefore, this study aims to evaluate the impact of central obesity severity on esophageal mucosa and esophageal motility function by calculating the V/S using conventional CT imaging.

## 2 Materials and methods

### 2.1 Study population and grouping

This study retrospectively collected data from patients who visited the Gastrointestinal Motility Center of the First Affiliated Hospital of Dalian Medical University between January 2020 and April 2025. All patients underwent fiberoptic gastroduodenoscopy, high-resolution manometry (HRM), 24-h pH-impedance monitoring, and upper abdomen or whole abdomen non-contrast CT. Inclusion criteria: (1) aged 18–90 years; (2) weight change not exceeding 5% within 3 months, no use of drugs affecting fat metabolism, and no participation in regular physical training; (3) complete clinical data. Exclusion criteria: (1) patients with a history of esophageal, gastric, or other abdominal tumors or surgeries; (2) patients with achalasia, peptic ulcers, or other diseases indicated by endoscopic examination; (3) patients with ineffective esophageal motility (IEM), esophagogastric junction outflow obstruction (EGJOO), or other esophageal motility disorders indicated by HRM; (4) patients with conditions that affect abdominal fat measurement, including history of major abdominal surgery or intra-abdominal adhesions; large abdominal space-occupying lesions, extensive ascites, or intestinal obstruction; severe hepatosplenic diseases, etc.; (5) radiotherapy, chemotherapy, etc. This study was approved by the Ethics Committee of the First Affiliated Hospital of Dalian Medical University (Approval No. PJ-KS-KY-2024-550).

### 2.2 Fiberoptic gastroduodenoscopy

All patients underwent a fiberoptic gastroduodenoscopy within 3 months prior to completing HRM and 24-h pH-impedance monitoring. The procedure was performed in strict adherence to international guidelines, and all examinations were conducted by experienced physicians.

### 2.3 High-resolution manometry

HRM using GAP-36A (Medkinetic Incorporated, Ningbo, China) was performed to evaluate esophageal functioning. Before undergoing the HRM, patients were instructed to stop taking proton pump inhibitor and prokinetic drugs for at least 14 days (20). Participants were required to fast for 8 h or more before the investigation. The catheter was inserted transnasally and navigated from the hypopharynx into the stomach. The tubes were adjusted for 3–5 min. Once the pressures of both the upper esophageal sphincter (UES) and LES stabilized, resting pressure was recorded for a minimum of 30 s. Patients were instructed to swallow 5 ml of water at room temperature every 30 s, completing this process more than 10 times overall. They were advised to avoid repeated swallowing, ensuring each swallow was distinct. Manoview version 3.0 software was employed for analysis, adhering to the Chicago Classification version 4.0 (21).

In this study, we incorporated several indicators to assess esophageal motility: LES resting pressure, residual pressure, length; UES resting pressure, residual pressure, length, distal contractile integral (DCI), distal latency (DL), intrabolus pressure (IBP), multiple rapid swallows distal contractile integral (MRS-DCI), number of peristaltic contractions, percentage of Ineffective Swallows and esophagogastric junction contractile integral (EGJ-CI).

### 2.4 24 -h pH-impedance monitoring

Twenty four hour pH-impedance testing was performed immediately after the HRM. A Sleuth VR Multichannel Intraluminal Impedance ambulatory system (Sleuth; Sandhill Scientific, Inc., Highlands Ranch, CO, United States) was used. A pH sensor was positioned 5 cm above the upper edge of the LES, a catheter was fixed at the nasal ala, and we recorded the start time. The patient was given instructions to fill in a monitoring diary accurately, including details such as the start and the end time of eating and lying down, the type of symptoms, and their respective start times. Patients were advised to follow their regular routine, reduce their intake of acidic food, beverages, and alcohol, avoid lying in bed all day, avoid strenuous exercise after a meal, and flush the catheter with warm boiled water. The catheter was removed after 24 h of monitoring. The monitor was connected to a computer, and analysis was conducted using specialized software (Bio View Analysis VR; Sand Hill Science, Inc.). The reduction of esophageal baseline impedance (BI) can reflect esophageal inflammation, and damage to the integrity of esophageal mucosa that does not show up in the endoscopic examination can be detected by this method. As swallowing and reflux activities during the day can affect the measurement of BI, the BI values were recorded when patients were asleep three different times, early in the morning (at 1, 2, and 3 a.m.). We took the average value of these as the mean nocturnal baseline impedance (MNBI) (22, 23). The study included mean nocturnal baseline impedance (MNBI) values for six channels (Z1–Z6) to evaluate the integrity of the esophageal mucosal barrier. Additionally, we incorporated the following indicators to assess esophageal reflux: acid exposure time (AET), DeMeester score (DMS), total reflux episodes, Mean Acid Clearance Time and bolus clearance time (BCT).

### 2.5 Measurement of abdominal fat

#### 2.5.1 CT acquisition

All abdomen CT examinations were conducted across multiple scanner platforms (Siemens Somatom Definition AS/Biograph 40, GE LightSpeed 8/VCT 64, or Philips Brilliance iCT 256) within. Imaging parameters were harmonized to: 5 mm slice thickness/spacing, 80–120 kVp tube voltage, and 500 mAs tube current.

#### 2.5.2 Measurement of VAT and SAT

**FIGURE 1:**
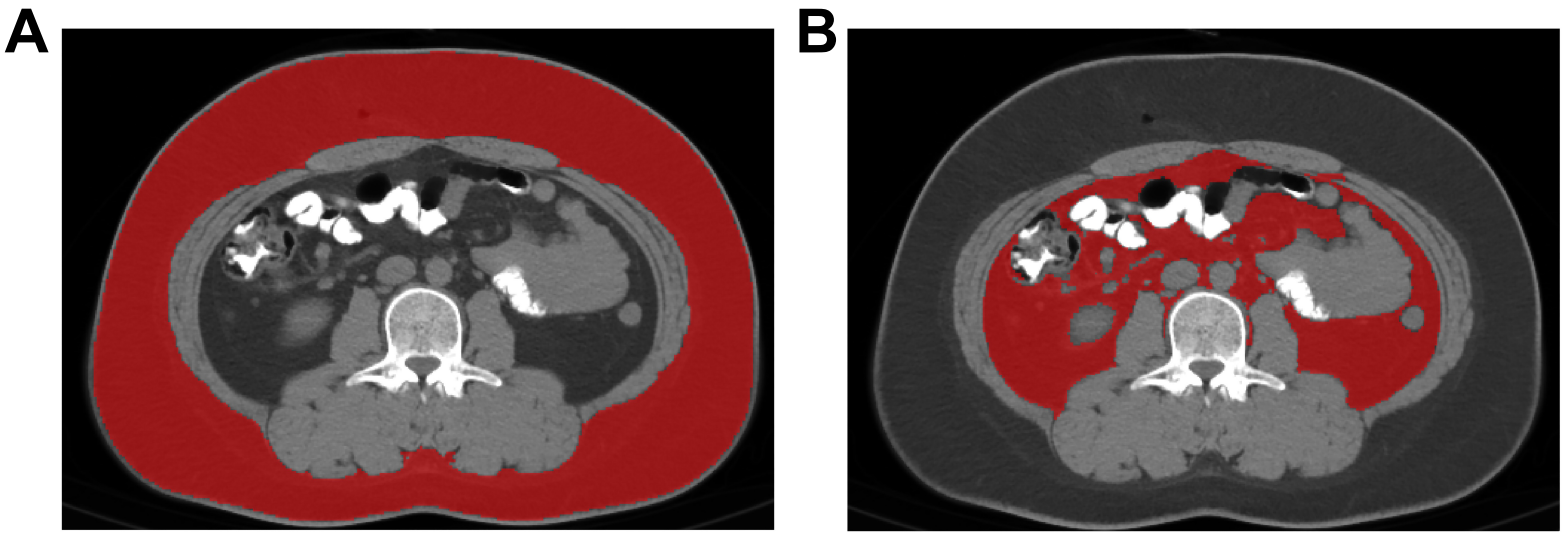
Segmentation images of SAT and VAT: **(A)** subcutaneous adipose tissue (SAT); **(B)** visceral adipose tissue (VAT).

All non-contrast abdomen CT images were stored in NIfTI format and processed using the open-source automatic segmentation tool Total Segmentator.^1^ First, intensity normalization and spatial normalization were performed on the images using this model to ensure effective processing of data acquired from different scanners and scanning parameters. Subsequently, the “tissue_types” task of Total Segmentator was employed to automatically segment SAT and VAT in CT images. Concurrently, the “total_vertebrae” task was utilized to segment vertebral bodies and automatically locate the 3rd lumbar vertebra (L3) (24), extracting segmentation images of SAT and VAT (Figures 1A,B). This study employed Python 3.9 programming language combined with customized computational scripts to process CT images for automated calculation of the area of SAT and VAT. Area calculation was achieved by iterating through each pixel in the image, counting the number of pixels belonging to a specific label, and multiplying by the pixel size to calculate the actual area. And the ratio of VAT area to SAT area is calculated to obtain V/S.Patients were grouped into quartiles according to the V/S ratios.

### 2.6 Statistical analysis

All statistical analyses and graphing in this study were performed using GraphPad Prism 9.5.0 software. The Kolmogorov-Smirnov test was used to verify the normal distribution of data. Comparisons of continuous variables among multiple groups were analyzed using one-way analysis of variance (ANOVA) for normally distributed data or the Kruskal-Wallis H test for skewed distributed data, with *P*-values adjusted for significance using the Bonferroni correction method for pairwise comparisons. Differences in categorical variables between groups were analyzed using the Chi-squared test. Continuous variables following a normal distribution were expressed as mean ± standard deviation ($\bar{x}$ s), while those with a skewed distribution were presented as median (P25, P75). Categorical variables were expressed as frequencies and percentages (n, %). Univariate regression analysis was performed using the Durbin-Watson test, and regression parameters were expressed as β-values. *P* < 0.05 was considered statistically significant.

## 3 Results

### 3.1 Patient characteristics

A total of 185 patients were enrolled in the study. All patients were grouped according to the quartiles of the V/S ratio, with the grouping results as follows: Q1 group: V/S ratio ≤ 1.01; Q2 group: 1.02 < V/S ratio < 1.29; Q3 group: 1.30 < V/S ratio < 1.75; Q4 group: V/S ratio ≥ 1.76.

The Q1 group consisted of 48 patients, including 8 males, with 15 cases of RE, 3 cases of BE, 15 cases of hiatal hernia (HH), 27 cases of EGJ type I, 8 cases of type II, and 13 cases of type III. The mean age was 52.75 years, and the median BMI was 24.30 kg/m^2^. The Q2 group included 45 patients, with 12 males, 13 cases of RE, 1 case of BE, 17 cases of HH, 26 cases of EGJ type I, 4 cases of type II, and 15 cases of type III. The mean age was 56.62 years, and the median BMI was 25.95 kg/m^2^. The Q3 group comprised 46 patients, with 23 males, 16 cases of RE, no cases of BE, 11 cases of HH, 27 cases of EGJ type I, 10 cases of type II, and 9 cases of type III. The mean age was 58.43 years, and the median BMI was 24.11 kg/m^2^. The Q4 group included 46 patients, with 30 males, 19 cases of RE, 2 cases of BE, 8 cases of HH, 33 cases of EGJ type I, 4 cases of type II, and 9 cases of type III. The mean age was 60.65 years, and the median BMI was 25.67 kg/m^2^. Results of 24-h pH-monitoring showed that the proportion of GERD among the four groups was 22.92, 31.11, 34.78, and 54.35%, with a significant difference in intergroup comparisons; gender also showed statistical significance in intergroup comparisons among the four groups (*P* < 0.05). Pairwise comparisons revealed that the proportion of male patients and the prevalence of GERD in the Q4 group were both higher than those in the Q1 group (Table 1).

**TABLE 1:** Comparison of baseline characteristics and HRM parameters across groups based on V/S quartiles.

Factors	Q1 (*n* = 48)	Q2 (*n* = 45)	Q3 (*n* = 46)	Q4 (*n* = 46)	*P*
Sex					**<0.001**
Male, n (%)	8	12	23	30	
Female, n (%)	40	33	23	16	
Age, years	52.75 ± 15.25	56.62 ± 17.04	58.43 ± 12.57	60.65 ± 12.38	0.057
RE, n (%)	15	13	16	19	0.623
Barrett’s esophagus, n (%)	3	1	0	2	0.359
HH, n (%)	15	17	11	8	0.148
GERD, n (%)	11	14	16	25	**0.012**
EGJ Type					0.421
Type I	27	26	27	33	
Type II	8	4	10	4	
Type III	13	15	9	9	
BMI, kg/m^2^	24.30 (20.38, 27.65)	25.95 (23.74, 28.67)	24.11 (20.91, 27.15)	25.67 (23.01, 27.48)	0.186
LES					
Resting pressure	8.00 (5.20, 11.00)	11.00 (7.75, 17.00)	13.70 (9.68, 19.25)	17.70 (11.73, 21.00)	**<0.001**
Residual pressure	3.05 (0.23, 5.90)	2.50 (0.40, 4.00)	3.05 (0.60, 7.00)	3.85 (2.28, 5.50)	0.179
Length	3.35 (2.85, 3.66)	3.18 (2.78, 3.74)	3.53 (2.80, 3.78)	3.30 (2.94, 3.75)	0.751
UES					
Resting pressure	30.85 (21.28, 44.95)	32.00 (23.05, 54.60)	30.90 (16.98, 39.90)	31.45 (19.65, 47.83)	0.545
Residual pressure	6.40 (3.63, 9.10)	7.80 (6.35, 11.35)	7.50 (4.60, 9.93)	8.05 (5.00, 11.13)	0.068
Length	3.79 (3.55, 4.39)	3.66 (3.55, 4.13)	3.61 (3.54, 4.25)	3.69 (3.55, 4.09)	0.644
DL	7.37 (6.31, 8.15)	7.09 (6.33, 7.78)	7.74 (6.62, 8.69)	7.18 (6.28, 8.20)	0.310
DCI	905.95 (506.5, 1484.05)	1104.90 (713.20, 1903.15)	1234.50 (426.10, 2187.75)	1192.50 (470.25, 2244.53)	0.438
IBP	6.55 (4.40, 13.60)	8.00 (4.10, 11.35)	9.40 (4.35, 13.60)	7.45 (3.98, 12.38)	0.889
MRSDCI	1.03 (0.64, 1.39)	0.80 (0.38, 1.23)	0.90 (0.46, 1.42)	1.04 (0.38, 1.29)	0.531
Number of peristaltic contractions	7.0 (4.0, 9.0)	8.0 (5.5, 9.0)	7.0 (2.75, 9.25)	9.0 (3.0, 10.0)	0.427
Percentage of Ineffective Swallows (%)	30.00 (0.00,60.00)	30.00 (10.00,50.00)	25.00 (0.00,70.00)	10.00 (0.00,62.50)	0.562
EGJ—CI	27.30 (14.60, 39.75)	35.10 (22.85, 51.95)	42.42 (22.70, 61.88)	49.20 (28.53, 67.23)	**<0.001**

RE, Reflux Esophagitis; HH, Hiatal Hernia; GERD, Gastroesophageal Reflux Disease; EGJ, esophagogastric junction; BMI, Body Mass Index; LES, Lower Esophageal Sphincter; UES, Upper Esophageal Sphincter; DL, distal latency; DCI, distal contractile integral; IBP, intrabolus pressure; MRSDCI, multiple rapid swallows distal contractile integral; EGJ-CI, esophagogastric junction contractile integral. Bold: *p*-value <0.05 was considered statistically significant.

### 3.2 Comparison of esophageal manometry indicators among four groups

**FIGURE 2:**
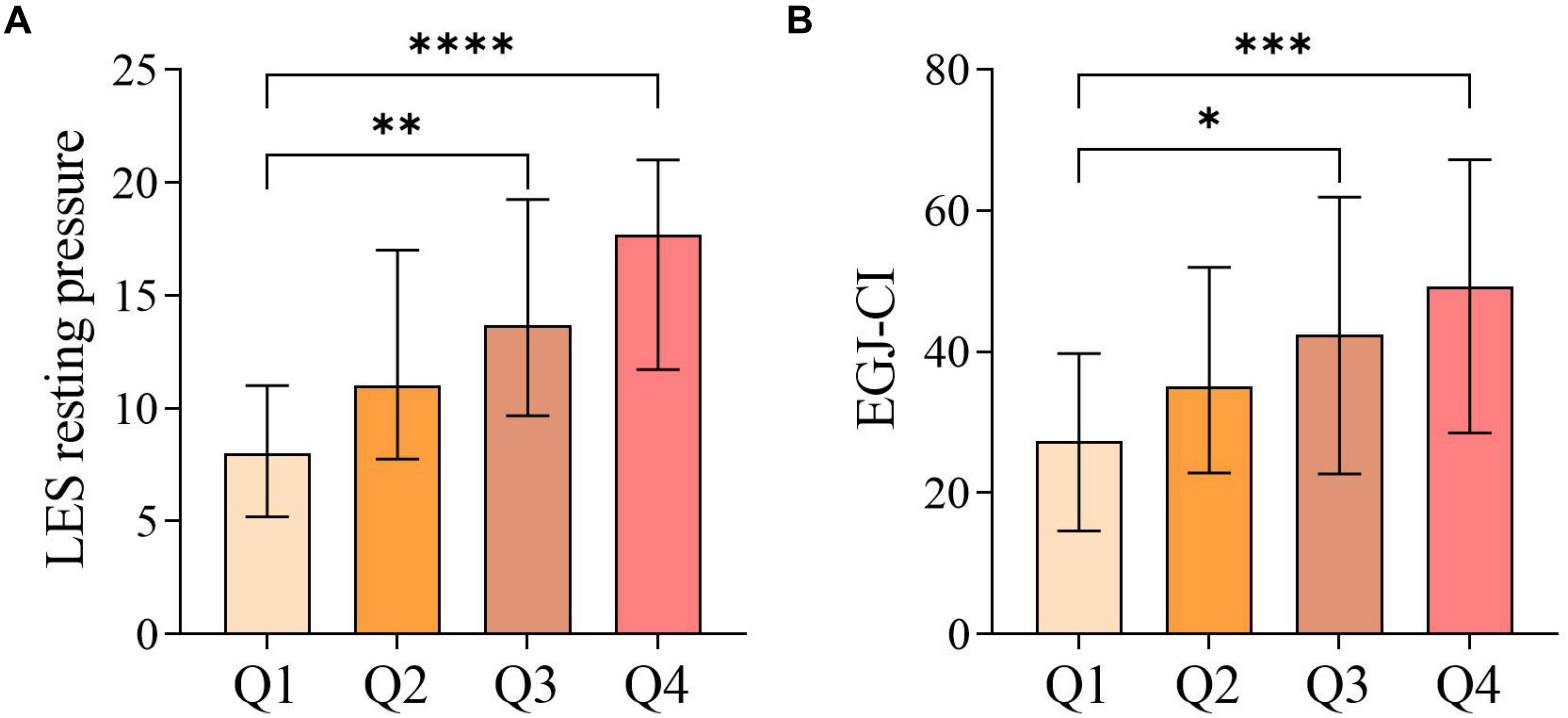
Pairwise comparison of esophageal manometry parameters among the four groups divided by V/S quartiles: **(A)** comparison of LES resting pressure; **(B)** comparison of EGJ-CI. *p < 0.05, **p < 0.01, ***p < 0.001, ****p < 0.0001.

Intergroup comparisons of HRM parameters among the four groups revealed that the LES resting pressure and EGJ-CI were statistically significant (*P* < 0.05) (Table 1). Further pairwise comparisons showed that both the LES resting pressure and EGJ-CI in the Q3 and Q4 groups were significantly higher than those in the Q1 group (*P* < 0.05), while no statistically significant differences were observed in other pairwise comparisons (Figures 2A,B).

### 3.3 Comparison of 24-h pH-impedance parameters and MNBI among the four groups

**FIGURE 3:**
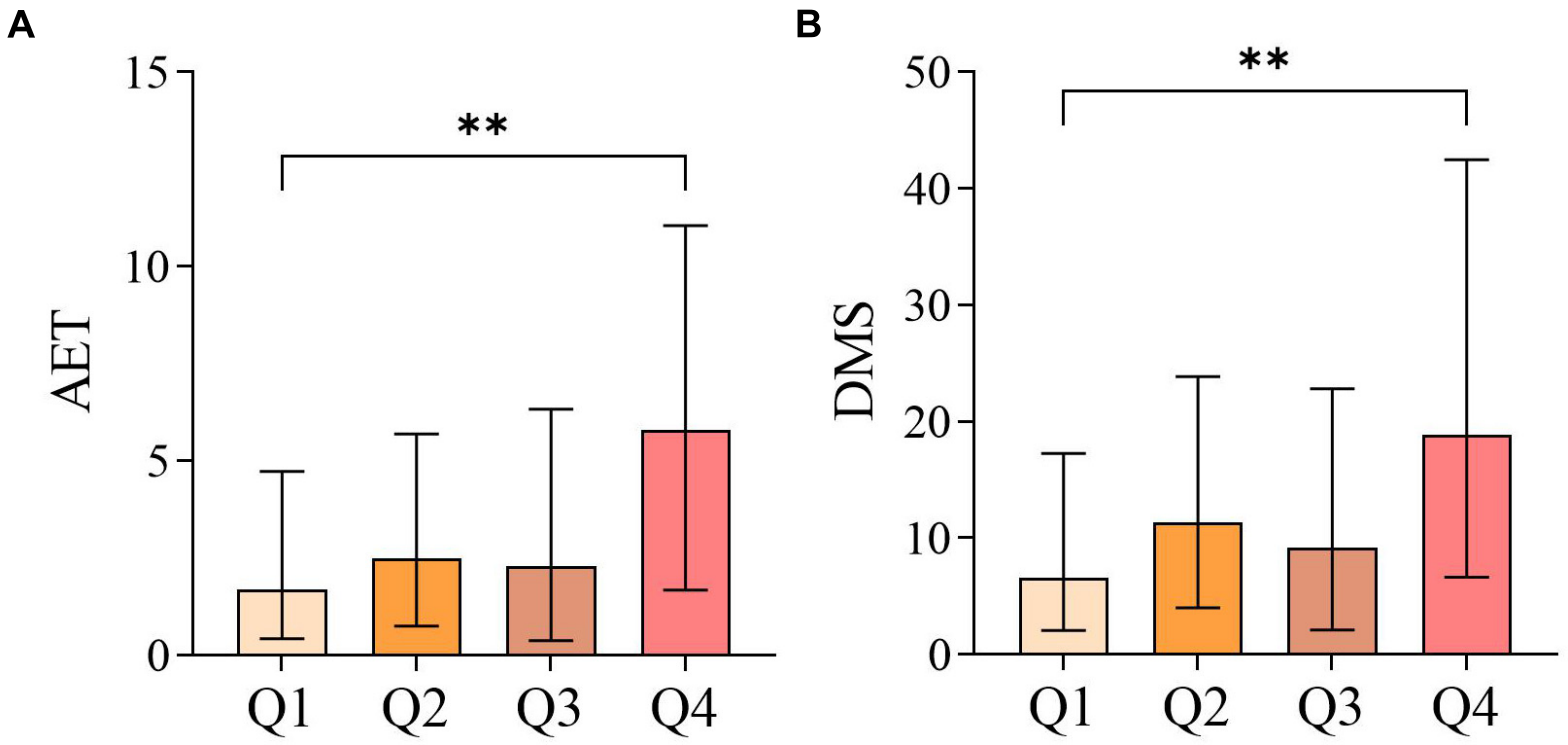
Pairwise comparison of 24-h pH-impedance parameters among the four groups divided by V/S quartiles: **(A)** comparison of AET; **(B)** comparison of DMS. *p < 0.05, **p < 0.01, ***p < 0.001, ****p < 0.0001.

Intergroup comparisons of 24-h pH-impedance monitoring parameters among the four groups showed that AET and DMS were statistically significant (*P* < 0.05). Further pairwise comparisons revealed that both AET and DMS in the Q4 group were significantly higher than those in the Q1 group (*P* < 0.05), while no statistically significant differences were observed in other pairwise comparisons (Figures 3A,B).

**FIGURE 4:**
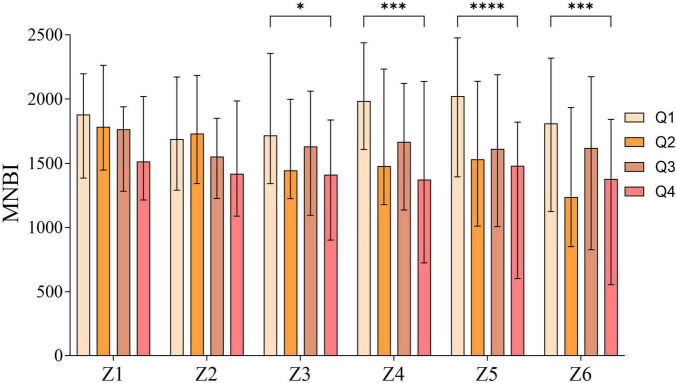
Pairwise comparison of MNBI values among the four groups divided by V/S quartiles. *p < 0.05, **p < 0.01, ***p < 0.001, ****p < 0.0001.

Among the six channels of MNBI, no statistically significant differences were observed in channels Z1 and Z2 among the four groups (*P* > 0.05), while significant differences were detected in channels Z3–Z6 (*P* < 0.05). Pairwise comparisons revealed that the MNBI values of channels Z3-Z6 in the Q4 group were significantly lower than those in the Q1 group (*P* < 0.05), with no statistically significant differences noted in other pairwise comparisons (Figure 4 and Table 2).

**TABLE 2:** Comparison of 24-h pH-impedance monitoring parameters and MNBI across groups based on V/S quartiles.

Factors	Q1 (*n* = 48)	Q2 (*n* = 45)	Q3 (*n* = 46)	Q4 (*n* = 46)	*P*
AET	1.70 (0.43, 4.73)	2.50 (0.75, 5.70)	2.30 (0.38, 6.33)	5.80 (1.68, 11.05)	**0.005**
DeMeester score	6.60 (2.08, 17.28)	11.30 (4.00, 23.85)	9.20 (2.10, 22.83)	18.90 (6.65, 42.48)	**0.007**
Total reflux episodes	24.00 (13.00, 39.25)	24.00 (14.50, 47.00)	27.50 (14.00, 44.50)	25.50 (14.00, 45.25)	0.827
Mean Acid Clearance Time	51.00 (25.25, 92.50)	66.00 (38.00, 91.50)	69.00 (33.25, 89.25)	75.00 (56.00, 106.75)	0.058
BCT	11.00 (8.00, 14.00)	11.00 (9.00, 13.00)	12.00 (8.00, 14.00)	11.50 (9.00, 14.00)	0.758
MNBI					
Z1	1880.50 (1384.50, 2196.75)	1785.00 (1447.50, 2263.00)	1765.00 (1282.25, 1940.75)	1515.00 (1214.75, 2021.00)	0.099
Z2	1688.00 (1290.25, 2172.25)	1732.00 (1341.50, 2184.50)	1553.00 (1226.25, 1850.75)	1419.50 (1088.25, 1986.25)	0.118
Z3	1717.00 (1342.25, 2355.75)	1445.00 (1225.50, 1998.50)	1632.50 (1094.25, 2062.50)	1411.50 (903.00, 1838.00)	**0.029**
Z4	1985.00 (1608.00, 2438.25)	1478.00 (1178.50, 2234.50)	1667.50 (1136.00, 2121.75)	1372.50 (724.75, 2138.00)	**0.007**
Z5	2024.00 (1394.00, 2476.75)	1532.00 (1011.00, 2138.50)	1613.50 (1007.75, 2189.50)	1482.00 (602.25, 1820.50)	**<0.001**
Z6	1810.50 (1125.00, 2318.75)	1238.00 (850.50, 1934.50)	1618.50 (826.75, 2174.25)	1379.50 (555.25, 1842.75)	**0.011**

AET, acid exposure time; BCT, Bolus Clearance Time; MNBI, mean nocturnal baseline impedance. Bold: *p*-value <0.05 was considered statistically significant.

### 3.4 Regression analysis between V/S and different indicators of esophageal functioning

To further explore the correlation between the aforementioned indicators and changes in the V/S, we performed a regression analysis of the V/S with these indicators based on the entire dataset.

Regression analysis showed that V/S was significantly correlated with LES resting pressure (β: 4.925, 95% CI: 3.310–6.539), EGJ-CI (β: 14.881, 95% CI: 9.663–20.099), AET (β: 2.537, 95% CI: 0.773–4.300), and DMS (β: 8.018, 95% CI: 1.954–14.082) (All *P* < 0.05). V/S was also significantly correlated with MNBI values in channels Z3 (β: −211.760, 95% CI: −371.965 to −51.555), Z4 (β: −270.759, 95% CI: −435.964 to −105.533), Z5 (β: −303.315, 95% CI: −470.376 to −136.254), Z6 (β: −256.112, 95% CI: −428.419 to −83.806) (All *P* < 0.05) (Table 3).

**TABLE 3:** Regression analysis of V/S and parameters of esophageal motility and mucosal barrier function.

Factors	β	95 %CI	*P-*value
LES resting pressure	4.925	3.310–6.539	**<0.001**
EGJ-CI	14.881	9.663–20.099	**<0.001**
AET	2.537	0.773–4.300	**0.005**
DeMeester score	8.018	1.954–14.082	**0.010**
MNBI			
Z3	−211.760	−371.965 to −51.555	**0.010**
Z4	−270.759	−435.964 to −105.533	**0.001**
Z5	−303.315	−470.376 to −136.254	**<0.001**
Z6	−256.112	−428.419 to −83.806	**0.004**

LES, Lower Esophageal Sphincter; EGJ-CI, esophagogastric junction contractile integral; AET, acid exposure time; MNBI, mean nocturnal baseline impedance. Bold: *p*-value <0.05 was considered statistically significant.

After adjusting for Mean Acid Clearance Time and BCT, V/S showed significant positive linear correlations with AET (β: 2.173, 95% CI: 0.704–3.643) and DMS (β: 6.766, 95% CI: 1.702–11.829), and a significant negative linear correlation with MNBI Z3-Z6. After adjusting for EGJ type and HH, V/S showed significant positive linear correlations with LES resting pressure (β: 4.437, 95% CI: 2.941–5.933) and EGJ-CI (β: 13.415, 95% CI: 8.468–18.363) (Supplementary Tables 1–3).

### 3.5 Correlation analysis between esophagus motility, reflux coefficients, and MNBI

**FIGURE 5:**
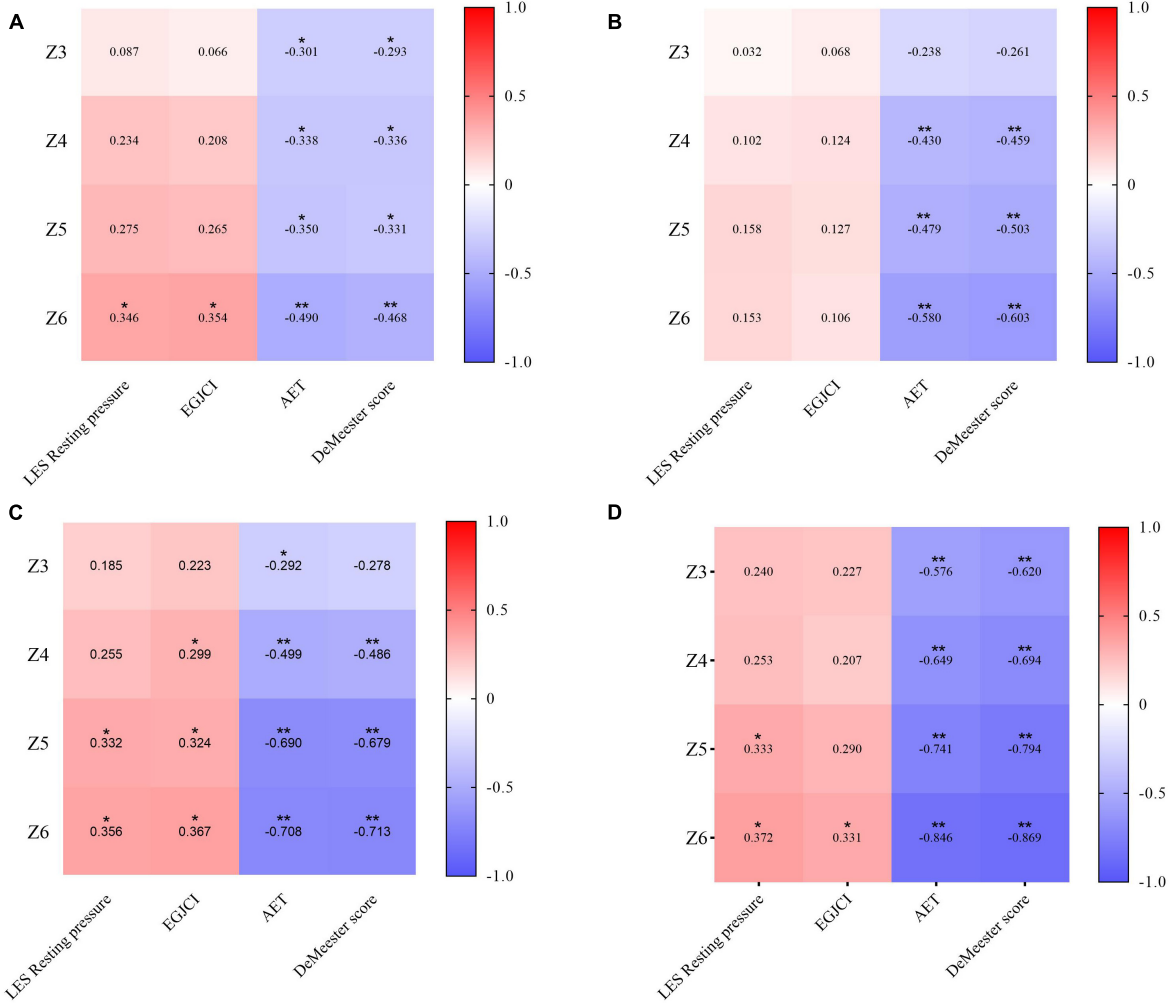
Correlation analysis between esophagus motility, reflux coefficients, and MNBI: **(A)** correlation analysis of Q1 group; **(B)** correlation analysis of Q2 group; **(C)** correlation analysis of Q3 group; **(D)** correlation analysis of Q4 group. *p < 0.05, **p < 0.01, ***p < 0.001, ****p < 0.0001.

Subsequently, a correlation analysis was conducted on the MNBI scores, esophageal manometry indicators, and reflux indices among the four groups of patients. The results showed that in the Q1 group, the decrease in MNBI values in channels Z3–Z6 is associated with acid reflux, and the decrease in MNBI at channel Z6 was associated with LES resting pressure and EGJ-CI; in the Q2 group, the decrease in MNBI values in channels Z4–Z6 is associated with acid reflux; in the Q3 group, the decrease in MNBI values in channels Z3–Z6 is associated with acid reflux, the decrease in MNBI at channel Z5–Z6 was associated with LES resting pressure and EGJ-CI, and the decrease in MNBI at channel Z4 was associated with EGJ-CI; in the Q4 group, the decrease in MNBI at channels Z3–Z6 was more strongly associated with acid reflux compared to the other groups, the decrease in MNBI values in channel Z6 was also associated with LES resting pressure and EGJ-CI, and the decrease in MNBI values in channel Z5 was also related to LES resting pressure (Figures 5A–D).

## 4 Discussion

In this study, patients’ CT imaging data, HRM, and 24-h pH-impedance monitoring parameters were retrospectively collected. The volumes of abdominal VAT and SAT were automatically quantified using CT, and the V/S was calculated to assess the severity of central obesity. Patients were grouped according to the V/S to investigate the impact of central obesity on esophageal function. The results of this study demonstrated that central obesity was associated to the increase esophageal pressure, impaired esophageal motility function, damaged esophageal mucosa, and weakened esophageal mucosa barrier function. With the progression of central obesity, esophageal mucosal injury is associated with reduced LES function, EGJ function, and acid reflux, with the correlation between esophageal mucosal injury and acid reflux becoming progressively stronger.

Obesity has been confirmed as a risk factor for RE and can also induce esophageal dysfunction. The incidence of primary esophageal motor disorder in the severely obese population is as high as 61%, which is significantly higher than that in the non-obese population (18). An increase in BMI is associated with an elevated prevalence of gastroesophageal reflux symptoms and complications of GERD (25, 26), and can also induce damage to the esophageal mucosa and impairment of esophageal motor function (27). Yet, other studies have indicated that neither body weight nor BMI is significantly correlated with 24-h acid reflux, upright/supine acid reflux (28). Mathus et al. (29) conducted 24-h monitoring on patients with morbid obesity who had a BMI of no less than 55 kg/m^2^, and found that morbid obesity had no significant impact on acid reflux. BMI can only reflect the degree of general obesity and is unable to further assess central obesity characterized by VAT accumulation. This may explain why most studies have not found an association between BMI and GERD diagnosed based on symptomatic criteria or indicators from 24-h pH-impedance monitoring (30). Furthermore, studies have indicated that central obesity characterized by increased VAT has a stronger correlation with RE. This is attributed to VAT’s high metabolic activity, as it can secrete pro-inflammatory cytokines, leading to systemic metabolic dysfunction. Abdominal fat also includes SAT; yet, SAT has low metabolic activity and serves as a neutral fat storage depot. Evidence suggests that SAT is not significantly associated with metabolic diseases or cardiovascular diseases, and acts as a protective fat reservoir in obese individuals with hypertriglyceridemia (31). Therefore, the impact of central obesity on esophageal function may vary with its severity. In this study, we quantified abdominal fat distribution using the V/S to investigate the effects of central obesity on esophageal function.

HRM is currently a widely used diagnostic tool for assessing esophageal motility function globally, and numerous studies have utilized this technique to explore the association between obesity and GERD. Yang et.al (27) assessed patients’ obesity status using BMI and found that the LES resting pressure, UES residual pressure, and EGJ-CI in patients with a BMI ≥ 25 kg/m^2^ were significantly higher than those in normal-weight individuals. Additionally, all three indicators showed a significant correlation with BMI. The results of LES resting pressure and EGJ-CI in this study were consistent with those findings. These two indicators increased with the elevation of the V/S (i.e., increased VAT or decreased SAT) across the four groups. Notably, there was no statistically significant difference in BMI among the four groups, which suggests that the confounding effect of BMI can be excluded. This further confirms that the core factor influencing esophageal function is central obesity rather than general obesity. Pandolfino et al. (32) also confirmed that esophageal pressure is higher in obese individuals, and that abdominal fat accumulation is a key factor contributing to elevated intraesophageal pressure. Increased accumulation of visceral adipose tissue can elevate intra-abdominal pressure, thereby increasing intraesophageal pressure (33, 34). Previous studies on healthy volunteers have shown that when intra-abdominal pressure is increased by wearing a belt, the abdominal segment of the LES is affected by the elevated intra-abdominal pressure, leading to a synchronous increase in its pressure (35). However, other studies have indicated that the LES pressure in obese individuals is significantly lower than that in normal-weight individuals (36). This may be attributed to the chronic effects of long-term elevated intra-abdominal pressure on the anatomical structure or function of the sphincter. Obesity can induce phospholipid metabolic imbalance by disrupting lipid metabolism, thereby reducing the binding efficiency of lysophosphatidic acid (LPA) receptors on the myocyte membrane of the LES. This impairs the regulatory effect of LPA on the contraction of sling fibers and clasp fibers, ultimately compromising the function of the intrinsic muscular layer of the LES (37, 38). In addition, the level of cholecystokinin (CCK) is significantly elevated in obese individuals, and CCK can induce LES relaxation by activating inhibitory motor neurons (39). Collectively, the aforementioned mechanisms can ultimately lead to a negative correlation between LES pressure and BMI.

The elevation of LES pressure may be associated with the reflex contraction of the crural diaphragm (CD). Increased intra-abdominal pressure (e.g., due to belt compression or leg raising) can induce this contraction, thereby leading to a subsequent rise in LES pressure (40). The Chicago Classification version 4.0 proposes that the LES and CD form the EGJ, which constitutes the antireflux barrier. The EGJ-CI serves as a valid indicator for assessing both the barrier function and contractile function of the EGJ. Specifically, lower EGJ pressure is associated with a lower EGJ-CI value (21, 41). Patients with EGJ types II and III demonstrated significantly lower EGJ-CI values than those with EGJ type I and exhibited a higher prevalence of HH (42, 43). To account for potential confounding by EGJ type and HH, regression analyses examining the relationship between V/S and both LES resting pressure and EGJ-CI were adjusted for these variables. The results indicated that the associations between V/S and EGJ function remained unaffected by the presence of HH or EGJ type. Studies have confirmed that EGJ pressure is positively correlated with intragastric pressure, and this correlation is particularly significant in EGJ type I and type II. This suggests that the elevation of EGJ pressure may be an adaptive response to increased intragastric pressure (44). Excessive accumulation of VAT in the abdominal cavity can elevate intra-abdominal pressure, which in turn leads to an increase in intragastric pressure and ultimately results in a higher EGJ-CI. In this study, with the increase in the V/S, the upward trend of EGJ-CI was particularly significant in the Q3 and Q4 groups. Sustained mechanical pressure exerted by VAT on esophageal smooth muscle induces the secretion of cytokines, which recruit mast cells and trigger local immune inflammation. In turn, inflammatory factors lead to abnormal smooth muscle contraction and impaired relaxation function through paracrine signaling, thereby reducing the compliance of the esophageal sphincter (45). Wu et al. (46) observed a significant correlation between VAT and elevated maximum standardized uptake values in the mid-esophagus and EGJ through positron emission tomography-CT examinations. This is consistent with the results of the regression analysis in this study, where a significant linear relationship was observed between the V/S and both LES resting pressure and EGJ-CI. Specifically, as the V/S increases, LES resting pressure and EGJ pressure increase accordingly.

Esophageal motility function was assessed using HRM. 24-h pH-impedance monitoring was employed to evaluate patients’ reflux status and esophageal mucosal barrier function, with the primary outcome measure for reflux assessment being AET. The Lyon Consensus specifies that an AET < 4% can be definitively classified as normal (physiological), while an AET > 6% is considered abnormal. Values falling between these two thresholds are categorized as an indeterminate range. Another outcome metric of pH-impedance monitoring is the number of reflux episodes, The Lyon GERD Consensus proposes that > 80 reflux episodes per 24 h are definitively abnormal, while a number < 40 is physiological (47). The DMS is an indicator that signals the presence of reflux, serving as a comprehensive scoring metric for acid exposure during long-term ambulatory pH monitoring. Its normal reference value is < 14.72, while a score ≥ 14.72 indicates pathological acid exposure in the esophagus. A key advantage of this score lies in its ability to fully reflect multiple characteristics of acid reflux, encompassing not only the single time proportion but also critical information such as reflux frequency and duration (48). BI values serve as a marker for esophageal mucosal integrity, with significantly lower values observed in both erosive and non-erosive GERD patients (49, 50). Reduced BI of the esophageal mucosa is associated with alterations in the intercellular spaces and tight junctions of esophageal mucosal cells (51, 52). Frequent swallowing and reflux events can interfere with the measurement of baseline impedance, BI is best measured from pH-impedance tracings during sleep, termed MNBI when averaged from three 10 min periods spaced an hour apart (53). In this study, there were statistically significant differences in AET and DMS among the four groups. Specifically, the AET and DMS in the Q4 group were significantly higher than those in the other three groups, with a significant difference only observed between the Q1 and Q4 groups in pairwise comparisons. This suggests that increased V/S is more likely to be accompanied by pathological acid reflux. Previous studies have confirmed that increased BMI and VAT accumulation are associated with an elevated risk of GERD (54, 55). BMI exhibits a linear correlation with esophageal acid exposure, and this association is mediated by the mechanical effect of increased intra-abdominal pressure (56). In the regression analysis of this study, a linear relationship was identified between the V/S and both AET and DMS, indicating that the level of acid reflux increases with the severity of central obesity. Additionally, this study compared MNBI values among the four groups and found statistically significant differences in the Z3-Z4 channels. Specifically, MNBI values in the Z3-Z4 channels showed a decreasing trend with increasing V/S, highlighting differences in esophageal mucosal function among patients with varying VAT contents. In pairwise comparisons, statistically significant differences in the Z3-Z4 channels were only observed between the Q1 and Q4 groups. Furthermore, MNBI values in the Z3-Z6 channels of both the Q2 and Q4 groups were less than 1,500 ohms. According to the Lyon Consensus 2.0, an MNBI value below 1,500 ohms indicates impaired esophageal mucosal integrity (57). To account for the potential influence of esophageal acid clearance on the reduction of MNBI, our study further adjusted for Mean Acid Clearance Time and BCT in the linear regression analysis of V/S and MNBI. The results demonstrated that the significant negative correlation between V/S and MNBI persisted even after this adjustment, suggesting that the decrease in MNBI associated with increased central obesity is independent of changes in esophageal acid clearance. Previous studies have confirmed that obesity independently reduces multi-channel MNBI values in the esophagus, independent of gastroesophageal reflux (58). Gibbens et al. (5) demonstrated that central obesity impairs the structural and functional integrity of the esophageal barrier, and this impairment is independent of increased esophageal acid exposure. It is characterized by intercellular space dilation, reduced desmosomal density, and increased fluorescein leakage in the distal esophagus. Robertson et al. (59) demonstrated that central obesity exacerbates the metaplasia of esophageal mucosal squamous epithelium to columnar epithelium, further leading to the expansion of cardiac mucosa. Cardiac mucosa is a type of non-acid-secreting columnar epithelial mucosa located between the esophageal squamous epithelium and the gastric acid-secreting epithelium. As a pathological lesion, it arises from the metaplasia of squamous epithelium in the most distal esophagus and can progress to esophageal adenocarcinoma (60). This study demonstrates that VAT accumulation can induce esophageal mucosal injury. However, aligning with previous literature (61), our study assessed the impact of central obesity on esophageal function using the V/S ratio, for which a standardized diagnostic cut off has yet to be established. Consequently, to refine the assessment of central obesity’s role in esophageal pathophysiology, future large-scale or multicenter trials are warranted to define a population-specific V/S threshold, particularly for the Chinese population.

The regression analysis results of this study showed that LES resting pressure, EGJ-CI, AET, DMS, and the values of the Z3–Z6 channels in MNBI were all statistically significant (*P* < 0.05). The results indicated that from a functional perspective, an increase in the V/S may affect LES pressure, the barrier function of the EGJ, the level of acid reflux, and esophageal mucosal integrity. To further clarify whether esophageal mucosal injury caused by different V/S levels is related to changes in esophageal motility and the level of acid reflux, we further performed correlation analysis on the aforementioned parameters among the four groups of patients. The results showed that esophageal mucosal injury in patients of the Q1 and Q2 groups was mainly related to acid reflux, and among them, mucosal injury in the Z6 channel of the Q1 group was also related to LES resting pressure; mucosal injury in patients of the Q3 and Q4 groups was similarly closely related to acid reflux, with a more significant correlation than that in the Q1 and Q2 groups, and was also related to esophageal motility abnormalities.

VAT exhibits high metabolic activity and can release pro-inflammatory cytokines such as free fatty acids, reactive oxygen species (ROS), tumor necrosis factor-α, and interleukin-6. These inflammatory factors inhibit the repair of esophageal mucosal cells, exacerbate esophageal injury by promoting oxidative stress, and disrupt the mucosal barrier (62, 63). Latest research has shown that the heat-labile substances secreted by the VAT at the EGJ induce the production of ROS, which in turn activates hypoxia—inducible factor-2α (HIF-2α) in esophageal squamous cells. HIF-2α interacts with the inflammasome–caspase-1 axis to promote the production of interleukin-1β (IL-1β). Subsequently, the IL-1β/IL-1R1 signaling pathway activates myosin light chain kinase, leading to myosin phosphorylation and triggering cytoskeletal contraction. Eventually, this results in dilated intercellular spaces and impairs the esophageal mucosa as well as its barrier function. Meanwhile, this study confirms that central obesity can disrupt the integrity of the esophageal barrier even in the absence of reflux (64). Second, as VAT accumulates, intra-abdominal pressure increases, leading to an elevation in gastroesophageal reflux. Prolonged exposure of the esophageal mucosa to gastric juice containing corrosive and irritant components further impairs the mucosal barrier (65). In this study, increased V/S ratio was associated with higher LES resting pressure, EGJ-CI, AET, DMS, and a greater prevalence of GERD. Physiologically, higher LES resting pressure and EGJ-CI enhance the esophageal anti-reflux barrier function, thereby reducing esophageal acid exposure and GERD prevalence (66). However, existing studies have shown that regardless of the presence of gastroesophageal reflux or increased acid exposure, central obesity can lead to esophageal functional impairment and an increased prevalence of GERD through the release of inflammatory factors and activation of inflammatory pathways (5, 64). Given that elevated AET and DMS may also reflect impaired esophageal acid clearance, we further incorporated Mean Acid Clearance Time and BCT to assess esophageal acid clearance function (67). No statistically significant differences were found in these acid clearance parameters across V/S groups. Furthermore, after adjusting for these parameters in linear regression models, V/S remained significantly and linearly correlated with both AET and DMS. These findings suggest that the increases in AET and DMS induced by central obesity may be independent of acid clearance function.

Central obesity may affect esophageal function through both metabolic and mechanical mechanisms. Based on the findings of this study, we proposed that the metabolic mechanism of central obesity on esophageal function might play a predominant role than the mechanical mechanism. As this hypothesis is derived from clinical observations, further mechanistic studies are warranted to validate these findings and elucidate the underlying biological processes.

This study has several strengths. Firstly, CT was used for quantitative analysis of VAT and SAT, enabling objective assessment of central obesity severity. Secondly, multiple examination indicators were incorporated to explore the association between central obesity and esophageal function. Finally, further analyses deepened the understanding of the relationship between central obesity and esophageal function. Yet, this study has several limitations. First, as a retrospective study, the enrolled patients presented had relatively mild reflux symptoms, and the sample size of patients with morbid obesity was limited, which restricts the generalizability of the findings. Second, clinical symptoms such as reflux status and dysphagia could not be collected retrospectively, resulting in the absence of clinical symptom scores and only the results of auxiliary examinations were included. Finally, there is currently a lack of standardized cutoff values for V/S to assess the severity of central obesity, which limits the clinical applicability of this metric.

## 5 Conclusion

Central obesity determined by CT imaging is associated with LES pressure, EGJ function, and increased acid reflux. Central obesity can also lead to esophageal mucosal damage. Worsening obesity exacerbates these reflux issues and impairs esophageal motility. Thus, managing central obesity is crucial to maintaining esophageal integrity and function and reducing reflux risk.

## Data Availability

The original contributions presented in this study are included in this article/Supplementary material, further inquiries can be directed to the corresponding authors.

## Footnotes

1
https://github.com/wasserth/TotalSegmentator
