# Supplementary material for: Impact of central obesity on esophageal motility and mucosal barrier function based on conventional CT evaluation
Source: Front Med (Lausanne). 2026 Mar 24;13:1768926. doi: 10.3389/fmed.2026.1768926 (PMC13053494; doi:10.3389/fmed.2026.1768926)
Supplement: Supplementary file 1 [file Table_1.docx]

***Supplementary materials***

Table S1. Linear Regression Analysis of V/S with AET and DeMeester Score Adjusted for Mean Acid Clearance Time and BCT

|  | β | 95 %CI | P value |
| --- | --- | --- | --- |
| AET |  |  |  |
| V/S | 2.173 | 0.704 - 3.643 | **0.004** |
| BCT | 0.445 | 0.286 - 0.605 | **＜0.001** |
| **Mean Acid Clearance Time** | 0.022 | 0.016 - 0.029 | **＜0.001** |
| DeMeester score |  |  |  |
| V/S | 6.766 | 1.702 - 11.829 | **0.009** |
| BCT | 1.511 | 0.961 - 2.061 | **＜0.001** |
| **Mean Acid Clearance Time** | 0.077 | 0.053 - 0.100 | **＜0.001** |

AET = acid exposure time; V/S = visceral-to-subcutaneous adipose tissue ratio; BCT = **Bolus Clearance Time**
