# Supplementary material for: Impact of central obesity on esophageal motility and mucosal barrier function based on conventional CT evaluation
Source: Front Med (Lausanne). 2026 Mar 24;13:1768926. doi: 10.3389/fmed.2026.1768926 (PMC13053494; doi:10.3389/fmed.2026.1768926)
Supplement: Supplementary file 2 [file Table_2.docx]

***Supplementary materials***

Table S2. Linear Regression Analysis of V/S with MNBI Z3-Z6 Adjusted for Mean Acid Clearance Time and BCT

|  | β | 95 %CI | P value |
| --- | --- | --- | --- |
| MNBI Z3 |  |  |  |
| V/S | -188.419 | -344.120 - -32.718 | **0.018** |
| BCT | - | - | 0.068 |
| **Mean Acid Clearance Time** | -1.293 | -1.998 - -0.587 | **＜0.001** |
| MNBI Z4 |  |  |  |
| V/S | -245.212 | -397.383 - -93.041 | **0.002** |
| BCT | -28.963 | -45.494 - -12.432 | **＜0.001** |
| **Mean Acid Clearance Time** | -1.553 | -2.249 - -0.856 | **＜0.001** |
| **MNBI Z5** |  |  |  |
| V/S | -281.142 | -436.523 - -125.762 | **＜0.001** |
| BCT | -30.907 | -47.786 - -14.027 | **＜0.001** |
| **Mean Acid Clearance Time** | -1.375 | -2.086 - -0.664 | **＜0.001** |
| **MNBI Z6** |  |  |  |
| V/S | -244.578 | -406.075 - -83.082 | **0.003** |
| BCT | -40.304 | -57.848 - -22.760 | **＜0.001** |
| **Mean Acid Clearance Time** | -0.830 | -1.570 - -0.091 | **0.028** |

MNBI = mean nocturnal baseline impedance; V/S = visceral-to-subcutaneous adipose tissue ratio; BCT = **Bolus Clearance Time**
