# Supplementary material for: Impact of central obesity on esophageal motility and mucosal barrier function based on conventional CT evaluation
Source: Front Med (Lausanne). 2026 Mar 24;13:1768926. doi: 10.3389/fmed.2026.1768926 (PMC13053494; doi:10.3389/fmed.2026.1768926)
Supplement: Supplementary file 3 [file Table_3.docx]

***Supplementary materials***

Table S3. Linear Regression Analysis of V/S with LES Resting Pressure and EGJ-CI Adjusted for HH and EGJ Type

|  | β | 95 %CI | P value |
| --- | --- | --- | --- |
| LES Resting pressure |  |  |  |
| V/S | 4.437 | 2.941 - 5.933 | **＜0.001** |
| HH | -3.407 | -6.790 - -0.024 | **0.048** |
| EGJ Type | -1.819 | -3.585 - -0.053 | **0.044** |
| EGJ-CI |  |  |  |
| V/S | 13.415 | 8.468 - 18.363 | **＜0.001** |
| HH | -17.878 | -25.014 - -10.742 | **＜0.001** |
| EGJ Type | - | - | 0.423 |

**LES** = **Lower Esophageal Sphincter;** V/S = visceral-to-subcutaneous adipose tissue ratio; **HH** = **Hiatal Hernia; EGJ =** esophagogastric junction;
